# Persistent chronic respiratory symptoms despite TB cure is poorly correlated with lung function

**DOI:** 10.5588/ijtld.20.0906

**Published:** 2021-04-01

**Authors:** B. W. Allwood, M. Stolbrink, N. Baines, E. Louw, D. T. Wademan, A. Lupton-Smith, S. Nel, D. Maree, S. Mpagama, M. Osman, F. M. Marx, G. Hoddinott, M. Lesosky, J. Rylance, K. Mortimer

**Affiliations:** 1 Division of Pulmonology, Department of Medicine, Stellenbosch University & Tygerberg Hospital, Tygerberg, South Africa; 2 Institute of Infection, Veterinary and Ecological Sciences, University of Liverpool, Liverpool, UK; 3 Desmond Tutu TB Centre, Department of Paediatrics and Child Health, Faculty of Medicine and Health Sciences, Stellenbosch University, Cape Town, South Africa; 4 Department of Science and Innovation-National Research Foundation South African Centre for Excellence in Epidemiological Modelling and Analysis (SACEMA), Faculty of Science, Stellenbosch University, Stellenbosch, South Africa; 5 Department of Physical Medicine and Rehabilitation, School of Medicine, Faculty of Health Sciences, University of Namibia, Windhoek, Namibia; 6 Kibong’oto Infectious Diseases Hospital, Kilimanjaro, Tanzania; 7 Division of Epidemiology & Biostatistics, School of Public Health & Family Medicine, University of Cape Town, South Africa; 8 Lung Health Group, Malawi-Liverpool-Wellcome Trust Clinical Research Programme, Blantyre, Malawi; 9 Liverpool School of Tropical Medicine, Liverpool, UK

**Keywords:** post-TB, spirometry, restriction, physiology, obstruction

## Abstract

**BACKGROUND:**

Persistent respiratory symptoms and lung function deficits are common after patients with TB. We aimed to define the burden of post-TB lung disease (PTLD) and assess associations between symptoms and impairment in two high TB incidence communities.

**METHODS:**

This was a cross-sectional survey of adults in Cape Town, South Africa who completed TB treatment 1–5 years previously. Questionnaires, spirometry and 6-minute walking distance (6MWD) were used to assess relationships between outcome measures and associated factors.

**RESULTS:**

Of the 145 participants recruited (mean age: 42 years, range: 18–75; 55 [38%] women), 55 (38%) had airflow obstruction and 84 (58%) had low forced vital capacity (FVC); the mean 6MWD was 463 m (range: 240–723). Respiratory symptoms were common: chronic cough (*n* = 27, 19%), wheeze (*n* = 61, 42%) and dyspnoea (modified MRC dyspnoea score 3 or 4: *n* = 36, 25%). There was poor correlation between FVC or obstruction and 6MWD. Only low body mass index showed consistent association with outcomes on multivariable analyses. Only 19 (13%) participants had a diagnosis of respiratory disease, and 16 (11%) currently received inhalers.

**CONCLUSION:**

There was substantial burden of symptoms and physiological impairment in this “cured” population, but poor correlation between objective outcome measures, highlighting deficits in our understanding of PTLD.

## Abstract (FR)

**CONTEXTE:**

Des symptômes respiratoires persistants et des déficits de la fonction pulmonaire sont fréquents après une TB. Nous avons tenté de définir le poids de la maladie pulmonaire post-TB (PTLD) et d’évaluer les associations entre symptômes et troubles respiratoires, dans deux communautés à incidence élevée.

**MÉTHODES:**

Enquête transversale auprès d’adultes au Cap, Afrique du Sud. Ceux-ci ont rempli questionnaire relatif au traitement de la TB dans les 1–5 années précédentes. Les questionnaires, une spirométrie et un test de marche de 6 minutes (6MWD) ont été utilisés pour évaluer les relations entre mesures de résultats et facteurs associés.

**RÉSULTATS:**

Nous avons recruté 145 participants d’âge moyen 42 ans (fourchette 18–75) dont 55 femmes (38%) ; 55 (38%) avaient une obstruction des voies aériennes, 84 (58%) une diminution de la FVC (capacité vitale forcée) et le 6MWD moyen était de 463 m (fourchette 240–723). Les symptômes respiratoires ont été fréquents : toux chronique (*n* = 27, 19%), sifflement (*n* = 61, 42%) et dyspnée (score modifié de Medical Research Council de dyspnée 3 ou 4 (*n* = 36, 25%). Il y a eu une corrélation médiocre entre FVC ou obstruction et 6MWD. Seuls les patients ayant un indice de masse corporelle faible ont mis en évidence une association cohérente avec les résultats d’analyse multivariable. Seulement 19 (13%) participants ont eu un diagnostic de maladie respiratoire et 16 (11%) ont reçu des inhalateurs.

**CONCLUSION:**

Il y a un poids substantiel de symptômes et de handicap physiologique dans cette population « guérie », mais une corrélation médiocre entre les mesures de résultats objectives, ce qui met en lumière le déficit de notre compréhension de la PTLD.

TB is one of the most common respiratory infections, and South Africa has one of the highest TB incidence rates in the world—around 615 per 100,000 population.^1,2^ There is a growing understanding that the effects of TB do not end with the completion of anti-TB chemotherapy.^3,4^ Residual, post-TB lung disease (PTLD) includes fibrosis, lung volume loss, cavitation, pleural disease, gas trapping and bronchiectasis, and can manifest with varying severity.^5^ In the multinational burden of obstructive lung disease study, people who completed TB treatment had a two-fold higher risk of developing both obstructive and restrictive lung disease compared to the general population.^6^

There is currently little evidence about how PTLD phenotypes progress or relate to symptoms. A prospective study of 405 patients in Malawi found 44% with bronchiectasis and 9% with one destroyed lobe on high-resolution computed tomography scanning.^7^ After 1 year, almost a third reported residual respiratory symptoms, one in five had a decline in forced expiratory volume in 1 sec (FEV_1_)of ≥100 ml, and 16% reported at least one respiratory exacerbation. In a cross-sectional study in suburban Cape Town, South Africa, approximately half of participants who had completed TB treatment in the previous 5 years reported ongoing respiratory symptoms.^8^

Better understanding of the interplay between symptoms, physiology and physical performance is needed, as well as predictive models of who may develop symptoms or require treatment for those at highest risk of PTLD. This is most relevant in low-resource, high-burden settings such as sub-Saharan Africa, where healthcare resources and diagnostics are limited.

In the present study, we evaluated the burden of symptoms, spirometric and physiological impairment in a community-based population who had successfully completed TB treatment, and investigated the relationship between symptoms and lung function.

## METHODS

### Setting, participants and sampling

We conducted a cross-sectional study of adults who had successfully completed TB treatment more than 1 year but less than 5 years prior to study enrolment. Potential participants who had completed an episode of TB treatment between 2013 and 2017 were identified through the Ikhwezi and Ravensmead Clinic registers in suburban Cape Town, as described elsewhere,^8^ and invited to participate. We used electronic TB registers from both primary healthcare facilities to obtain a random sample of individuals who were recorded for treatment during this time frame, and with a documented standard treatment outcome of either “cure” or “treatment completed”. Study recruitment and follow-up occurred between October and December 2018 (Ikhwezi) and March and November 2019 (Ravensmead). Individuals were contacted during study visits at the home address recorded in the treatment register. Individuals were eligible for this study if they were at least 18 years old, had at least one episode of treated TB (as per list), and provided informed written consent. Exclusion criteria were pregnancy, currently receiving TB treatment, contra-indications to performing spirometry and acute illness (likely to introduce measurement error).

### Outcomes

Primary outcome was the prevalence of symptoms, physiological impairment and spirometric abnormalities. Secondary outcomes included correlation between the primary outcomes and a model for spirometric and 6-min walking distance (6MWD) results.

### Data collection

Participants completed the IMPALA (International Multidisciplinary Programme to Address Lung Health and TB in Africa) symptoms, smoking, environmental exposures, life exposures, previous episodes of TB and nutrition questionnaires.^9^ Data were collected electronically using KoBo Toolbox (Harvard Humanitarian Initiative, Cambridge, MA, USA).^10^ Breathlessness was rated using the modified Medical Research Council (mMRC) scale.^11^ Demographic data were captured and managed using REDCap (Vanderbilt University, Nashville, TN, USA) electronic data capture tools hosted at Stellen-bosch University, Tygerberg, South Africa.^12^ Spirometry and the 6-min walking test were conducted at the study locations. Spirometry was performed before and after inhaled salbutamol 400 mcg via a metered-dose inhaler using EasyOne^®^ (ndd Medical Technologies, Zurich, Switzerland) device according to the American Thoracic Society/European Respiratory Society guidelines. Post-bronchodilator spirometry data are presented here.Normal spirometric ranges were defined by GLI (Global Lung Function Initiative) 2012, and abnormal results were defined those less than the lower limit of normal (LLN) (*Z-*score <−1.64).^13^

### Statistical methods

Data were presented as means, standard deviations, medians, ranges or percentages based on the type and distribution of data. Pearson’s and Spearman’s correlation were used for the correlation analysis. Student’s *t*-test, analysis of variance (ANOVA), χ^2^ and Kruskal-Wallis tests were used for univariable analysis. Linear or logistic regression analysis was used for the multivariable analysis. Outcome variables were forced vital capacity (FVC) % predicted, 6-min walking distance (6MWD) and forced expiratory volume in 1 s (FEV_1_)/FVC < LLN. Variables were included in the model based on exposures of interest and possible confounders. Variables were retained to create the smallest Akaike Information Criterion (AIC) in the final backwards multivariable regression analysis. Significance was defined as *P* < 0.05, 95% confidence intervals (CIs) have been provided where appropriate. Data were analysed using R (R Computing, Vienna, Austria).^14^

### Ethical considerations

Ethical approval was obtained from Stellenbosch University, Tygerberg, South Africa; (N18/05/056) and the Liverpool School of Tropical Medicine, Liverpool, UK (18-050). Permission to conduct the study was obtained from the Western Cape Department of Health, Cape Town, South Africa; all participants provided written informed consent prior to participating in the study.

## RESULTS

### Descriptive data

**Figure 1:**
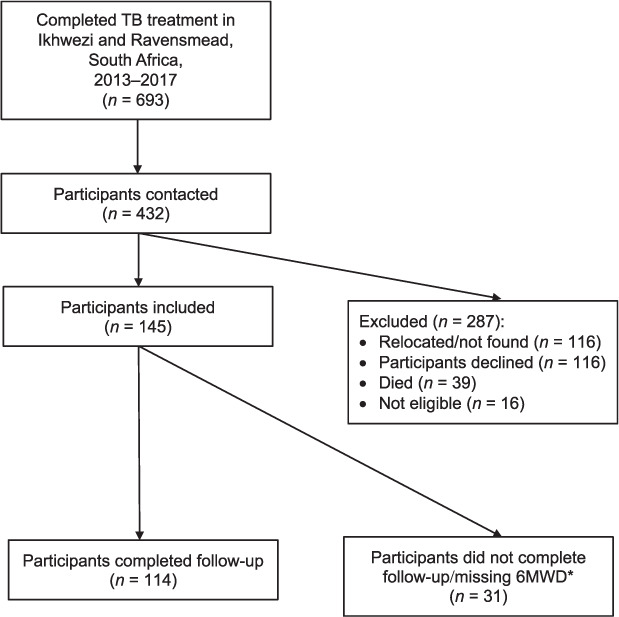
Flowchart of participant eligibility and inclusion into the study. *Participants did not have 6MWD due to delayed start of staff (n = 8), low resting peripheral saturations (<94%, n = 3), injury (n = 1), patient preference (n = 1), unknown (n = 18). 6MWD = 6-min walking distance.

A total of 432 participants from the registers were contacted, 39 had died (9.0%) and 277 were invited to take part (Figure 1). We recruited 145 study participants (Figure 1), of which 55 (38%) were women. The mean age was 42 years (range 18–75, Table 1). Over half of the participants left school at or before age 15 (66%). Of the 145 participants, 24 were living with HIV, 26 had hypertension and 19 diabetes; 19 had a respiratory disease diagnosis and 16 were prescribed inhalers. Cough, phlegm production, and wheeze were present in respectively 19%, 17% and 42% of participants; 55 (38%) had been treated more than once for TB, with a range of up to 6 times. Most participants reported having their first episode of TB as adults. Seven (5%) did not complete treatment on at least one occasion and 10 (7%) had been treated for multi- or extensively drug-resistant TB (MDR- and XDR-TB) at least once (Figure 1).

**Table 1:** Baseline participant characteristics

Patient characteristic	*n* (%)^*^
Sex	
Female	55 (38)
Male	90 (62)
Age, years, mean (range)	42 (18–75)
Highest level of education	
<Grade 10	73 (50)
Grade 10	23 (16)
Grade 10–12	33 (29)
Tertiary education	1 (1)
Unknown	5 (3)
Self-reported HIV status	
Negative	109 (75)
Positive (23 on ART)	24 (17)
Unknown	12 (8)
Comorbidities^†^	
Diabetes	19 (13)
Hypertension	26 (18)
Cardiac disease	4 (3)
Respiratory disease diagnosis	19 (13)
Asthma	14
Post-TB bronchiectasis and COPD	1
Other	4
Inhalers received	16 (11)
Asthavent (short-acting beta-agonist)	5
Asthavent + other	2
Other	9
Symptoms	
mMRC	
mMRC 0	82 (57)
mMRC 1	20 (14)
mMRC 2	7 (5)
mMRC 3	17 (12)
mMRC 4	19 (13)
Chronic cough for at least 3 months/year	27 (19)
Chronic phlegm for at least 3 months/year	24 (17)
Presence of wheezing attacks in the last 12 months	61 (42)
Number of wheezing attacks in the last 12 months, median (range) [IQR]	2 (1–12) [1–4]
Previous TB	
Number of times treated for TB	
1	90 (62)
2	38 (26)
3	10 (7)
4	4 (3)
5	2 (1)
6	1 (1)
Age at first TB, years, median (range) [IQR]	32 (8–70) [23–42]
Number of those ever having had less than 6 months of treatment	7 (5)
Drug susceptibility of previous TB episodes	
MDR/XDR-TB	10 (7)
Drug-susceptible TB	135 (93)
6MWT	
6MWT performed	114 (79)
6MWT distance, m, mean (range)	463 (240–723)
Spirometry	
FEV_1_, L, mean (range)	2.24 (0.40–4.67)
FEV_1_, % predicted, mean (range)	68 (20–117)
FVC, L, mean (range)	3.03 (0.65–5.50)
FVC, % predicted, mean (range)	75 (22–125)
Low FVC by LLN	84 (58)
FEV_1_/FVC ratio, median (range)	0.75 (0.30–1.00)
Obstruction by LLN	55 (38)
Smoking	
Smoking status	
Current	85 (59)
Ex	15 (10)
Never	45 (31)
Smoking duration, years, mean (range)	23 (1–55)
Smoking, pack years, median (range) [IQR]	6.7 (0.1–43) [3.5–13.3]
Smoked substances other than cigarettes	51 (35)
Other substances smoked (multiple answers possible)	
Cannabis	39
Waterpipe	12
Tik (crystal methamphetamine)	5
Mandrax (methaqualone)	8
Other	4
Exposure to air pollution	
Residing beside a major road	46 (32)
Prolonged (>15 h) exposure to vapours, dusts, gases or fumes at home or in workplace	90 (62)
Main mode of travel	
Walking	55 (38)
Minibus	36 (25)
Car	11 (8)
Not specified	43 (29)
Nutrition	
Ever experienced lack of food	82 (57)
Alcohol use	71 (49)
Units of alcohol consumed/week, median [IQR]	21 [7.5–38]
BMI, kg/m^2^, median [IQR]	20.7 [18.5–25.6]

^*^ Missing percentages due to rounding.

^†^ Participants can have multiple comorbidities.

ART = antiretroviral treatment; COPD = chronic obstructive pulmonary disease; mMRC = modified Medical Research Council; IQR = interquartile range; MDR-TB = multidrug-resistant TB; XDR-TB = extensively drug-resistant TB; 6MWT=6-min walking test; FEV_1_=forced expiratory volume in 1 sec; FVC = forced vital capacity; LLN = lower limit of normal; BMI = body mass index.

There was a high prevalence but low intensity of smoking: 100 (69%) reported current or previous smoking, but participants only accumulated a median of 6.7 pack-years; 51 (35%) participants reported smoking substances other than cigarettes, the most common being cannabis and waterpipe.

About one third lived close to roads (*n* = 46, 32%), and walking was the main mode of travel for 55 (38%). Prolonged exposure to dusts, gases and fumes was reported by 90 participants (62%); 82 (59%) reported having experienced lack of food and 71 (49%) consumed alcohol (median: 21 units/week, interquartile range [IQR] 7.5–38). The median body mass index (BMI) was 20.7 kg/m^2^ (IQR 18.5–25.6).

### Outcome data

Of the 145 participants, 82 (57%) reported minimal dyspnoea (mMRC 0), but 25% described significant breathlessness symptoms (mMRC 3 or 4, see Supplementary Table S1). The mean FEV_1_ was 2.24 L (range: 0.40–4.67), and a mean of 68% predicted FEV_1_. The mean FVC was 3.03 L, a mean of 75% predicted. The median FEV_1_/FVC ratio was 0.75, and 55 (38%) of participants had obstruction defined by the LLN. The mean 6MWD was 463 m (range: 240–723); 31 participants did not complete a 6-minute walk test (Table 1).

### Uni- and multivariable analysis

The main outcome variables were FVC (% predicted) to measure restriction, FEV_1_/FVC ratio as per the LLN (to measure obstruction) and 6MWD (as a physiological measure). For the univariable analysis, all participants with data for that variable were included. All those with fully completed data sets across all variables (*n* = 107) were included in the multivariable analysis.

There was no evidence of correlation between the three outcome variables: FVC vs. obstruction (point-biserial correlation coefficient −0.11, 95% CI −0.30 to 0.08), FVC vs. 6MWD (Pearson coefficient 0.07, 95% CI −0.11 to 0.25) and obstruction vs. 6MWD (point-biserial correlation coefficient −0.07, 95% CI −0.24 to 0.12), nor between FEV_1_ in participants with obstruction and 6MWD (see Supplementary Figures).

**Figure 2:**
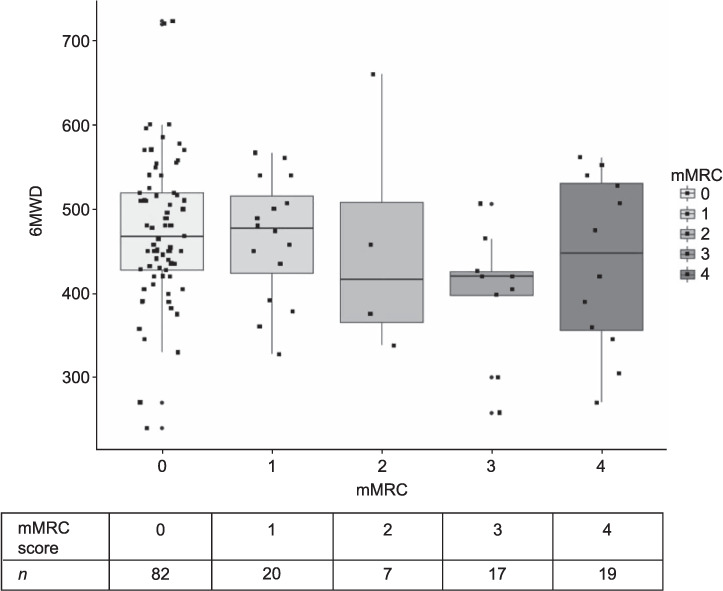
Boxplot of mMRC score vs. 6MWD (m). Boxplots show median, upper and lower quartiles, with total range and individual data points. 6MWD = 6-min walking distance; mMRC = modified Medical Research Council.

mMRC scores were not related to shorter 6MWD (one-way ANOVA *P* = 0.13; F(4,19) = 1.82). Those with mMRC scores of 2 or 3 appeared to have a reduced 6MWD, but this was not maintained for those with an mMRC score of 4 (Figure 2).

For the 84 participants with low FVC (*Z*-score <1.64 of normal), the mean 6MWD was 465 m compared to 458 m of patients with normal FVC (*P* = 0.69, 95% CI for difference of the mean: −43 to 28).^12^ The mean 6MWD in patients with airflow obstruction was 450 m, compared to 469 m in patients without airflow obstruction (*P* = 0.31, 95% CI for difference of mean −19 to 56).

FVC (% predicted), BMI, alcohol consumption and chronic bronchitis symptoms were statistically significantly associated in univariable analysis (Table 2). BMI, alcohol consumption and symptoms of chronic bronchitis were present in the final multivariable, adjusted regression model (*R*^2^ 0.14; AIC 594).

**Table 2:** Results of univariable and multivariable linear regression analysis for FVC (% predicted) ^*^

Variable	Univariable analysis		Multivariable analysis	
	
	Unadjusted coefficient estimate (95% CI)	*P* value	Adjusted coefficient estimate (95% CI)	*P* value
Male sex	−4.80 (−11.52 to 1.92)	0.16		
Age, years	−0.05 (−0.29 to 0.19)	0.70		
BMI	0.49 (0.00 to 1.00)	0.05	0.61 (0.13 to 1.10)	0.01^†^
Alcohol consumption	6.89 (0.53 to 13.25)	0.03^†^	9.22 (3.06 to 15.38)	<0.01^†^
Highest level of education^‡^	Linear coefficient: −2.29 (−8.81 to 4.24)	0.49		
	Quadratic coefficient: −3.57 (−10.69 to 3.56)	0.32		
	Cubic coefficient: −4.43 (−12.11 to 3.25)	0.26		
HIV status	Positive: 7.33 (−1.75 to 16.42)	0.11		
	Unknown: −2.26 (−15.36 to 10.84)	0.73		
Respiratory disease diagnosis	Yes: −9.61 (−21.86 to 2.64)	0.12		
	Unknown: 5.96 (−13.56 to 25.48)	0.55		
mMRC Score^‡^	Linear: −2.87 (−10.98 to 5.24)	0.48		
	Quadratic: 11.15 (−0.03 to 22.34)	0.05		
	Cubic: 1.57 (−8.55 to 11.69)	0.76		
6MWD, m	0.00 (−0.02 to 0.05)	0.62		
Chronic cough for at least 3 months per year	−8.88 (−17.82 to 0.06)	0.05		
Chronic bronchitis symptoms^§^	Present: −8.16 (−16.35 to 0.04)	0.05	−10.35 (−18.28 to −2.41)	0.01^†^
	Unknown: −12.37 (−29.20 to 4.46)	0.15	Unknown: −14.68 (−30.69 to 1.32)	0.07
Number of times treated for TB (1, 2, ≥3)	Linear coefficient: −1.50 (−9.55 to 6.55)	0.71		
	Quadratic coefficient: −2.67 (−9.64 to 4.30)	0.45		
Age at first TB, years	−0.02 (−0.27 to 0.22)	0.84		
Ever not completed treatment	−1.75 (−14.87 to 11.38)	0.79		
Ever had MDR/XDR-TB	−10.99 (−30.54 to 8.56)	0.27		
Smoking status	Ex: 3.03 (−10.31 to 16.37)	0.65		
	Never: 2.87 (−4.40 to 10.14)	0.44		
Ever smoked substance other than cigarettes	3.56 (−3.15 to 10.27)	0.30	5.06 (−1.33 to 11.44)	0.12
Any prolonged (>15 h) exposure to vapours, dusts, gases or fumes at home or in job	−0.78 (−7.61 to 6.04)	0.82		
Final model fit			Adjusted *R*^2^: 0.14	AIC: 594

^*^ Variables were included based on exposures of interest and possible confounders. Statistically significant variables as per final backwards multivariable regression analysis. For binary variables, the other group is the comparator.

^†^ *P* < 0.05.

^‡^ mMRC and education level treated as ordinal categorical values.

^§^ Defined as productive cough for at least 3 months in the previous 2 years.

FVC = forced vital capacity; CI = confidence interval; BMI = body mass index; mMRC = modified medical research council; 6MWD = 6-minute walking distance; MDR-TB = multidrug- resistant; XDR-TB = extensively drug-resistant TB; AIC = Akaike Information Criterion.

6MWD, age, BMI, highest level of education, HIV status, age at first TB, FEV_1_ (in L) and FVC (in L) were statistically significant in the univariable analysis (Table 3). The multivariable linear regression model included age, BMI, alcohol consumption, highest level of education, HIV status, chronic cough, chronic bronchitis, smoking status and smoking substances other than cigarettes (*R*^2^ 0.37; AIC 920).

**Table 3:** Results of univariable and multivariable linear regression analysis for 6-minute walking distance ^*^

Variable	Univariable analysis		Multivariable analysis	
	
	Unadjusted coefficient estimate (95% CI)	*P* value	Adjusted coefficient estimate (95% CI)	*P* value
Male sex	15.80 (−19.09 to 50.70)	0.37		
Age, years	−3.22 (−4.30 to −2.16)	<0.01^†^	−2.24 (−3.47 to 1.01)	<0.01^†^
BMI	−4.72 (−7.20 to −2.23)	<0.01^†^	−4.30 (−6.61 to −1.99)	<0.01^†^
Alcohol consumed	−16.84 (−50.25 to 16.56)	0.32	−23.30 (−51.05 to 4.45)	0.10
Highest level of education^‡^	Linear coefficient: 24.70 (−7.58 to 56.99)	0.13	Linear coefficient: −0.61 (−29.99 to 28.77)	0.97
	Quadratic coefficient: −29.01 (−64.27 to 6.25)	0.11	Quadratic coefficient: −2.41 (−36.16 to 31.34)	0.89
	Cubic coefficient: −48.18 (−86.19 to −10.18)	0.01^†^	Cubic coefficient: −52.95 (−86.33 to −19.57)	<0.01^†^
HIV status	Positive: 49.29 (2.73 to 95.85)	0.04^†^	Positive: 49.55 (9.31 to 89.79)	0.02^†^
	Unknown: 8.76 (−58.39 to 75.90)	0.80	Unknown: 13.16 (−42.76 to 69.08)	0.65
Respiratory disease diagnosis	Yes: −26.76 (−90.64 to 37.13)	0.41		
	Unknown 14.16 (−87.62 to 115.94)	0.78		
mMRC Score^‡^	Linear: −37.92 (−80.13 to 4.28)	0.08		
	Quadratic: 9.87 (−48.34 to 68.09)	0.74		
	Cubic: 19.16 (−33.51 to 71.83)	0.47		
Chronic cough for at least 3 months per year	−18.92 (−65.78 to 27.95)	0.43	78.01 (−4.72 to 160.74)	0.07
Chronic bronchitis symptoms^§^	Yes: −31.92 (−74.85 to 11.02)	0.14	Yes: −79.01 (−156.59 to −1.43)	0.05^†^
	Unknown: 8.06 (−80.18 to 96.30)	0.86	Unknown: 21.07 (−50.71 to 92.85)	0.57
Number of times treated for TB (1, 2, ≥3)	Linear coefficient: −5.72 (−47.38 to 35.94)	0.79		
	Quadratic coefficient: −6.94 (−43.00 to 29.12)	0.70		
Age at first TB, years	−2.89 (−4.01 to −1.77)	<0.01^†^		
Ever not completed treatment	−4.18 (−71.97 to 63.61)	0.90		
Ever had MDR/XDR-TB	47.09 (−54.05 to 148.22)	0.36		
Smoking status	Ex: 19.21 (−49.67 to 88.08)	0.58	Ex: 58.41 (1.16 to 115.66)	0.05
	Never: 14.28 (−23.26 to 51.28)	0.45	Never: 13.72 (−23.64 to 51.08)	0.47
Ever smoked substance other than cigarettes	25.75 (−8.72 to 60.23)	0.14	23.91 (−7.41 to 55.23)	0.14
Any prolonged (>15 h) exposure to vapours, dusts, gases or fumes at home or in job	−7.59 (−42.80 to 27.63)	0.67		
FEV_1_, L	35.00 (13.91 to 56.09)	<0.01^†^		
FVC, % predicted	0.25 (−0.75 to 1.24)	0.62		
Obstruction by LLN	−19.13 (−54.67 to 16.41)	0.29		
Final model fit			Adjusted *R*^2^: 0.37	AIC: 920

^*^ Variables were included based on exposures of interest and possible confounders. Statistically significant variables as per final backwards multivariable regression analysis. For binary variables, the other group is the comparator.

^†^ *P* < 0.05.

^‡^ mMRC and education level treated as ordinal categorical values.

^§^ Defined as productive cough for at least 3 months in the previous 2 years.

CI = confidence interval; BMI = body mass index; mMRC = modified medical research council; MDR-TB = multidrug-resistant TB; XDR-TB = extensively drug-resistant TB; FEV_1_ = forced expiratory volume in 1 sec; FVC = forced vital capacity; LLN = lower limit of normal; AIC = Akaike Information Criterion.

Spirometric obstruction was statistically significantly influenced only by BMI in the uni- and multivariable logistic regression analysis (Table 4). The final multivariable logistic regression model included BMI, chronic cough, chronic bronchitis, alcohol consumption and 6MWD. This model accounted for 70% of the variation in the data (AIC 128.3; Cox-Snell *R*^2^ 0.18; Nagelkerke *R* 0.25).

**Table 4:** Results of univariable and multivariable logistic regression analysis for spirometric obstruction (FEV_1_/FVC less than lower limit of normal) ^*^

Variable	Univariable analysis		Multivariable analysis	
	
	OR (95% CI)	*P* value	aOR (95% CI)	*P* value
Male sex	1.31 (0.56–3.17)	0.54		
Age, years	1.02 (0.99–1.05	0.31		
BMI	0.91 (0.83–0.99)	0.04^†^	0.88 (0.79–0.96)	0.01^†^
Alcohol consumed	0.67 (0.30–1.51)	0.34	0.85 (0.17–4.25)	0.07
Highest level of education^‡^	Linear: 0.53 (0.22–0.64)	0.14		
	Quadratic: 1.96 (0.72–6.13)	0.21		
	Cubic: 3.19 (1.04–12.71)	0.06		
HIV status	Positive: 1.34 (0.42–4.01)	0.61		
	Unknown: 1.67 (0.31–8.12)	0.52		
mMRC Score^‡^	Linear: 1.98 (0.68–5.63)	0.20		
	Quadratic: 0.39 (0.09–1.57)	0.17		
	Cubic: 0.39 (0.09–1.37)	0.16		
6MWD, m	1.00 (0.18–15.30)	0.29	0.99 (0.98–1.01)	0.91
Chronic cough for at least 3 months per year	0.92 (0.27–2.79)	0.89	0.00 (0–∞)	0.99
Chronic bronchitis symptoms^§^	Yes: 2.01 (0.73–5.49)	0.17	OR (yes): 0.00 (0.00–∞)	0.99
	Unknown: 2.46 (0.28–21.46)	0.38	OR (unknown): 3.76 (0.34–44.05)	0.26
Number of times treated for TB (1, 2, ≥3)	Linear: 1.98 (0.75–5.27)	0.16		
	Quadratic: 0.96 (0.42–2.24)	0.93		
Age at first TB, years	1.00 (0.97–1.03)	0.87		
Ever not completed treatment	1.59 (0.30–7.64)	0.56		
Ever had MDR/XDR-TB	4.30 (0.40–94.54)	0.24		
Smoking status	Ex: 0.75 (0.10–3.77)	0.74		
	Never: 0.77 (0.30–1.89)	0.57		
Ever smoked substance other than cigarettes	1.79 (0.78–4.13)	0.17		
Any prolonged (>15 h) exposure to vapours, dusts, gases or fumes at home or in job	0.59 (0.25–1.36)	0.21		
Final model			AIC: 128.3	
			Cox–Snell *R*^2^: 0.18	
			Nagelkerke: 0.25	

^*^ Variables were included based on exposures of interest and possible confounders. Statistically significant variables as per final backwards multivariable regression analysis. For binary variables, the other group is the comparator. No obstruction is comparator group for all ORs.

^†^ *P* < 0.05.

^‡^ mMRC and education level treated as ordinal categorical values.

^§^ Defined as productive cough for at least 3 months in the previous 2 years.

FEV_1_ = forced expiratory volume in 1 sec; FVC = forced vital capacity; OR = odds ratio; CI = confidence interval; aOR = adjusted OR; BMI = body mass index; mMRC = modified medical research council; 6MWD = 6-min walking distance; MDR-TB = multidrug-resistant TB; XDR-TB = extensively drug-resistant TB; AIC = Akaike Information Criterion.

## DISCUSSION

This cohort of 145 participants who had successfully completed TB treatment between 1 and 5 years previously had high burden of symptoms, short 6MWD and spirometric obstruction. We were unable to find strong associations between risk factors and symptoms, spirometric or physiological impairment, and there was poor correlation between outcome measures themselves (spirometric and functional).

Despite the high burden of respiratory abnormalities, only 19 participants had a formal diagnosis of respiratory disease and only 16 received inhalers at enrolment. Almost a third reported significant breathlessness (mMRC ≥2), more than 40% attacks of wheezing, and about 1 in 5 chronic cough and phlegm. It is surprising to note that 38% had chronic airflow obstruction and the mean 6MWD was 463 m, as this was a young community population. A hundred participants were current or ex-smokers; however, smoking intensity was low (median consumption: 6.7 pack years), implying cigarette smoke a less likely cause for symptoms. Smoking other substances and exposures to potentially damaging inhalants were common; however, neither these, nor cigarette smoking were associated with objective outcome measures. Seventeen participants reported at least three previous episodes of TB and most first contracted TB as middle-aged adults, but the number of TB episodes, drug resistance and age of first episode were not associated with outcomes, as has been suggested elsewhere.^15^

A very high prevalence of spirometric obstruction has been described in previous multi-centre, multinational studies.^16^ A similar study in rural South Africa found comparatively more preserved lung function and fewer symptoms but much reduced 6MWD (mean: 294 m) compared to our population.^17^ A Malawian cohort demonstrated lower symptom burden than our population, with 12% having symptom-related impairment in their ability to work 1 year after treatment completion, with a mean 6MWD of 611 m.^7^ Whether there is an effect of urbanisation on PTLD needs to be established.

The associations between various commonly reported objective measures of respiratory function were poor. This adds complexity to the syndrome of PTLD, which is known for its heterogeneity, and amplifies the difficulty in the epidemiological measurement and modelling of disability in PTLD. The persistent symptomatology observed and the well-described excess mortality (9% of our sample population had died) cannot be ignored;^18,19^ however, our knowledge of the interplay between spirometry, symptoms and physiology in PTLD is incomplete. Studies of PTLD in Mexico and the United States described a negative correlation between spirometry abnormalities and symptoms.^20,21^ These correlations have also been investigated in other respiratory conditions, such as asthma, with inconsistent results.^22–27^ In chronic obstructive pulmonary disease (COPD), dyspnoea has been established as a marker for mortality, with mMRC scores of 3 or 4 associated with a relative risk of death of 8.3 and 61 respectively.^28^ These conflicting data indicate that for now we should continue investigating PTLD patients multi-dimensionally until better outcome data are available.

The models derived suggest some factors may be associated with the spirometry, physiological and symptomatic impairments described. Chronic cough or chronic bronchitis symptoms appeared in final models for 6MWD and FVC but not spirometric obstruction, and could be explored as one potential screening tool for impairment. Low BMI and alcohol use were frequently associated, but the reason for the association is unclear. Both could be confounders, associated with a common unknown independent variable, for example, poor nutrition or poor social circumstances, or both could be effect modifiers in the pathway to PTLD, or potentially even a consequence (rather than a cause) of chronic lung impairment. The usual cautions need to be employed when interpreting cross-sectional associations and attributing either effect modification or prediction to the outcome.

Although participants were assessed in detail regarding their symptoms, spirometry and functional capacity, we did not consider their radiological findings. Chest X-ray is likely to be a less sensitive measure of post-TB impairment than spirometry, as it cannot identifypredominantly small-airway PTLD.^29^ The baseline data reflect the population sampled—limited education, almost 20% HIV prevalence, with an experience of food shortages and a low–normal BMI, so the high burden of observed PTLD could have particularly significant economic, morbidity and mortality implications in this vulnerable population.

Clearly there is a need for a better understanding of the pathophysiology of PTLD and the relationship between objective physiological measures, symptoms and meaningful outcomes such as mortality, exacerbations and morbidity (including psychosocial impacts). It is likely that a multimodal approach will be needed, analogous to that used for other respiratory diseases, for example, the BODE (Body-mass index, airflow Obstruction, Dyspnea, and Exercise) index in COPD.^30,31^ Developing such a tool for PTLD would be helpful to risk stratify patients in limited healthcare settings such as sub-Saharan Africa. Although self-selection through participation and the sample size may have impacted our study findings, unlike previous studies of PTLD, patients were not actively healthcare-seeking and were selected at random from a TB register between 1 and 5 years after treatment completion. These high rates of residual abnormalities after successful treatment, combined with low rates of chronic respiratory diagnosis or treatment, again highlights the need to look beyond usual outcome measures in TB trials and control programmes. Given the significant symptoms and functional impairment in this young population, urgent prognostic assessments for PTLD are needed, and pulmonary rehabilitation programmes may play a significant role in its treatment.^32–35^ More work is required on the long-term evolution of PTLD to identify individuals at risk of chronic PTLD and to investigate effective prevention and treatment strategies.

## CONCLUSION

This population exhibited a high burden of self-reported symptoms, airflow obstruction and functional impairment 1–5 years after TB treatment completion, yet only a minority had received a diagnosis of chronic disease or were receiving treatment. There was poor correlation between physiology, functional capacity and symptoms, with only low BMI most consistently associated with outcomes. This highlights the need for a better understanding of disease pathophysiology and multimodal assessments to determine meaningful objective measurements in PTLD. More work is needed on the prognosis of and interventions for PTLD, considering that more than 58 million people have survived TB in the last two decades alone.

## References

[1] World Health Organization. (2019). TB profile: South Africa. https://worldhealthorg.shinyapps.io/tb_profiles/?_inputs_&lan=%22EN%22&iso2=%22ZA%22.

[2] TBfacts.org. (2020). TB facts: information about TB. https://tbfacts.org/.

[3] Tiberi S (2019). Managing severe tuberculosis and its sequelae: From intensive care to surgery and rehabilitation. J Bras Pneumol.

[4] Allwood BW (2020). Post-tuberculosis lung health: perspectives from the First International Symposium. Int J Tuberc Lung Dis.

[5] Meghji J (2016). A systematic review of the prevalence and pattern of imaging defined post-TB lung disease. PLoS One.

[6] Amaral AFS (2015). Tuberculosis associates with both airflow obstruction and low lung function: BOLD results. Eur Respir J.

[7] Meghji J (2020). Patient outcomes associated with post-tuberculosis lung damage in Malawi: a prospective cohort study. Thorax.

[8] Osman M (2019). Morbidity and mortality up to 5 years post tuberculosis treatment in South Africa: A pilot study. Int J Infect Dis.

[9] Rylance J (2020). IMPALA Questionnaires. https://github.com/jipp3r/IMPALA_QuestionSet.

[10] KoBoToolbox. (2020). https://www.kobotoolbox.org/.

[11] Hajiro T (1998). Analysis of clinical methods used to evaluate dyspnea in patients with chronic obstructive pulmonary disease. Am J Respir Crit Care Med.

[12] Harris PA (2009). Research electronic data capture (REDCap)—A metadata-driven methodology and workflow process for providing translational research informatics support. J Biomed Inform.

[13] Quanjer PH (2012). Multi-ethnic reference values for spirometry for the 3-95-yr age range: The global lung function 2012 equations. Eur Respir J.

[14] Core Team. (2019). R: a language and environment for statistical computing. https://www.r-project.org/.

[15] Choi H (2014). Predictors of pulmonary tuberculosis treatment outcomes in South Korea: A prospective cohort study, 2005–2012. BMC Infect Dis.

[16] Lamprecht B (2011). COPD in never-smokers: results from the population-based BOLD Study. Chest.

[17] Daniels KJ (2019). Post-tuberculosis health-related quality of life, lung function and exercise capacity in a cured pulmonary tuberculosis population in the Breede Valley District, South Africa. S Afr J Physiother.

[18] Miller TL (2015). Mortality hazard and survival after tuberculosis treatment. Am J Public Health.

[19] Fox G (2019). Post-treatment mortality among patients with tuberculosis: a prospective cohort study of 10 964 patients in Vietnam. Clin Infect Dis.

[20] Pasipanodya JG (2007). Using the St. George Respiratory Questionnaire to ascertain health quality in persons with treated pulmonary tuberculosis. Chest.

[21] Báez-Saldaña R (2013). A novel scoring system to measure radiographic abnormalities and related spirometric values in cured pulmonary tuberculosis. PLoS One.

[22] Carter R (2003). 6-minute walk work for assessment of functional capacity in patients with COPD. Chest.

[23] Karanth MS, Awad NT (2017). Six minute walk test: a tool for predicting mortality in chronic pulmonary diseases. J Clin diagnostic Res.

[24] Camargo L, Pereira C (2010). Dyspnea in COPD: Beyond the modified Medical Research Council scale. J Bras Pneumol.

[25] Bijl-Hofland ID (1999). Perception of bronchoconstriction in asthma patients measured during histamine challenge test. Eur Respir J.

[26] Grazzini M (2001). Relevance of dyspnoea and respiratory function measurements in monitoring of asthma: A factor analysis. Respir Med.

[27] Cowie RL, Underwood BMF, Field SK (2007). Asthma symptoms do not predict spirometry. Can Respir J.

[28] Nishimura K (2002). Dyspnea is a better predictor of 5-year survival than airway obstruction in patients with COPD. Chest.

[29] Stek C (2020). The effect of HIV-associated tuberculosis, tuberculosis-IRIS and prednisone on lung function. Eur Respir J.

[30] Celli BR (2004). The body mass index, airflow obstruction, dyspnea, and exercise capacity index in chronic obstructive pulmonary disease. N Engl J Med.

[31] Chong WF (2004). The body mass index, airflow obstruction, dyspnea, and exercise capacity index in predicting hospitalization for chronic obstructive pulmonary disease. Chest.

[32] Muñoz-Torrico M (2020). Functional impact of sequelae in drug-susceptible and multidrug-resistant tuberculosis. Int J Tuberc Lung Dis.

[33] Muñoz-Torrico M (2016). Is there a rationale for pulmonary rehabilitation following successful chemotherapy for tuberculosis?. J Bras Pneumol.

[34] Visca D (2020). The need for pulmonary rehabilitation following tuberculosis treatment. Int J Tuberc Lung Dis.

[35] Akkerman OW (2020). Rehabilitation, optimized nutritional care, and boosting host internal milieu to improve long-term treatment outcomes in tuberculosis patients. Int J Infect Dis.

